# Community-acquired Pneumonia with Methicillin-resistant Staphylococcus Aureus in a Patient Admitted to the Intensive Care Unit: A Therapeutic Challenge

**DOI:** 10.7759/cureus.2019

**Published:** 2018-01-03

**Authors:** John Pham, Talal Asif, Majdi S Hamarshi

**Affiliations:** 1 Department of Internal Medicine, University of Missouri Kansas City (UMKC); 2 Department of Critical Care Medicine, Saint Lukes Hospital, University of Missouri Kansas City (UMKC)

**Keywords:** community acquired mrsa, linezolid, mrsa

## Abstract

Methicillin-resistant S*taphylococcus aureus* (MRSA) was previously considered a purely nosocomial pathogen. However, community-acquired MRSA has recently emerged as an important cause of severe necrotizing community-acquired pneumonia (CA-MRSA) in previously healthy individuals. This new pathogen exhibits antibiotic resistance and is linked to extended hospital stay and higher mortality. CA-MRSA has presented new therapeutic challenges due to high vancomycin treatment failure and lack of specificity of clinical findings. There is emerging evidence that treatment with linezolid leads to better patient outcomes in patients with CA-MRSA. Through this case, we aim to raise awareness about early institution of therapy for CA-MRSA whenever it is suspected, to improve patient outcomes.

## Introduction

Linezolid and vancomycin are both recommended for treatment of methicillin-resistant *Staphylococcus aureus* (MRSA) pneumonia by most clinical societies. Some prospective randomized trials showed that the clinical and microbiological response of hospital or ventilator-associated pneumonia might be more favorable with linezolid as compared to vancomycin [1]. Case reports, experts, and at least one randomized clinical trial also suggest using linezolid in community-acquired pneumonia secondary to MRSA [1, 2]. Linezolid’s possible superiority over vancomycin stems from the fact that it has better penetration into lung parenchyma, better availability at the tissue level, predictable blood level with kidney disease, and inhibition of toxin production in toxin-producing strains as in community-acquired MRSA (CA-MRSA) [1]. This case report describes the clinical course of a patient with community-acquired pneumonia secondary to toxin-producing CA-MRSA.

## Case presentation

**Figure 1:**
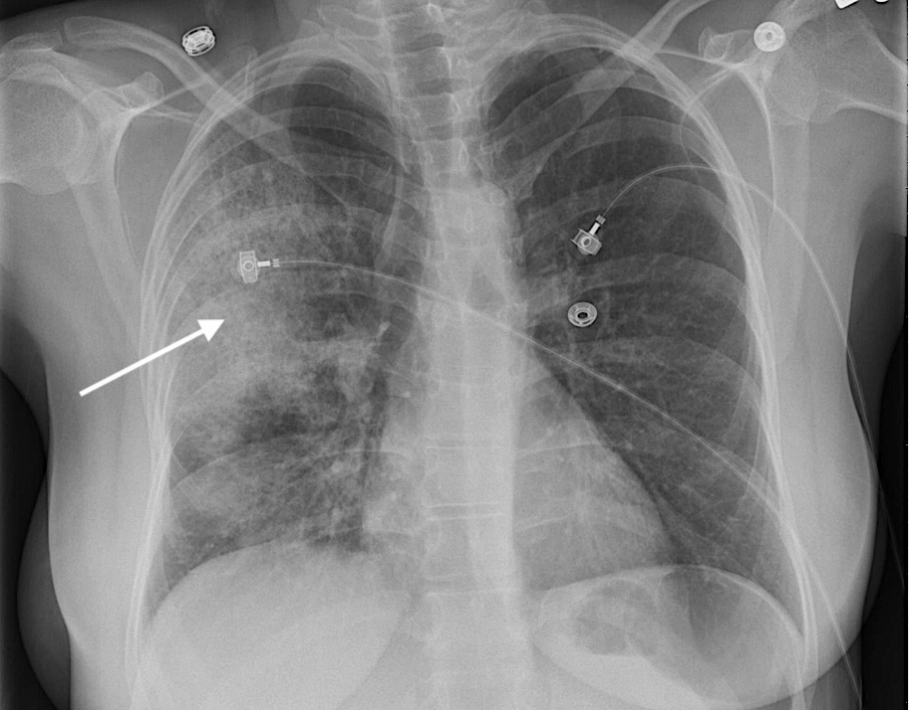
Patient's initial chest X-ray with arrow depicting right upper lobe pneumonia.

A 41-year-old female patient with a past medical history of bipolar disorder, cocaine use disorder, and tobacco use disorder, presented with increased respiratory distress and combativeness. On initial examination, her vital signs included a temperature of 100.9°F, heart rate of 140 beats per minute, respiratory rate of 30 breaths per minute and blood pressure of 103/66 mmHg. On physical examination, she was only able to speak in two-word sentences. There were bilateral coarse crackles all over the chest. She did not have any focal neurological deficits. Cardiovascular examination did not reveal abnormal heart sounds. Abdominal examination was unremarkable. The patient was immediately placed on a nonrebreather mask. However, the patient kept taking off her nonrebreather mask, continued to have worsening hypoxia, and eventually required tracheal intubation. Chest radiograph showed right upper lobe pneumonia (Figure 1).

**Figure 2:**
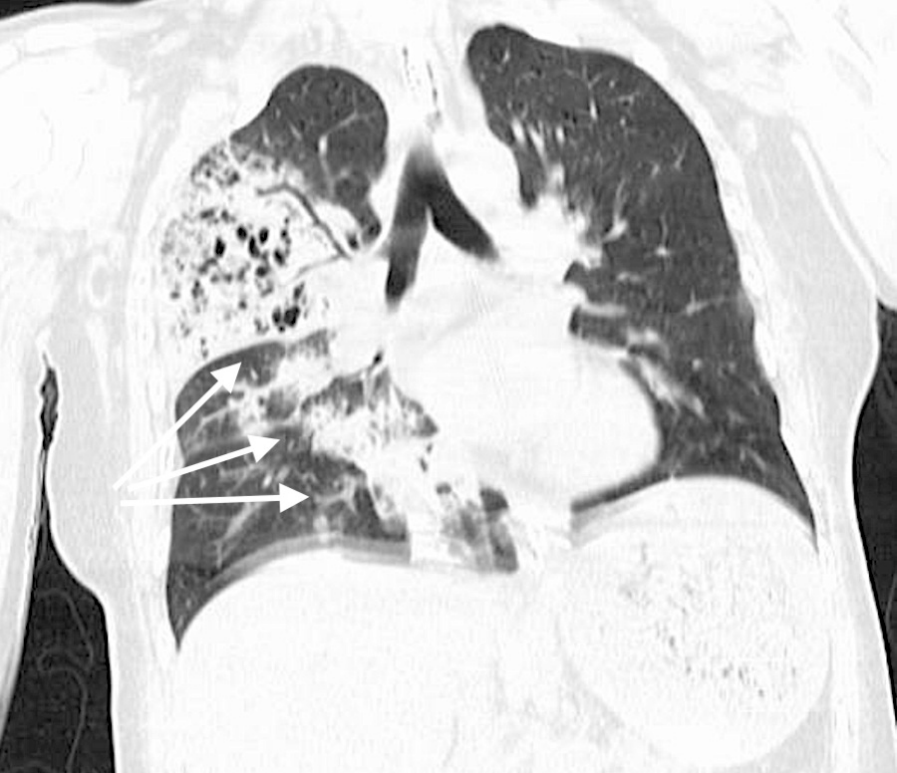
Computed tomography (CT) chest (coronal view) showing right upper, middle, and lower lobe consolidations (arrows).

**Figure 3:**
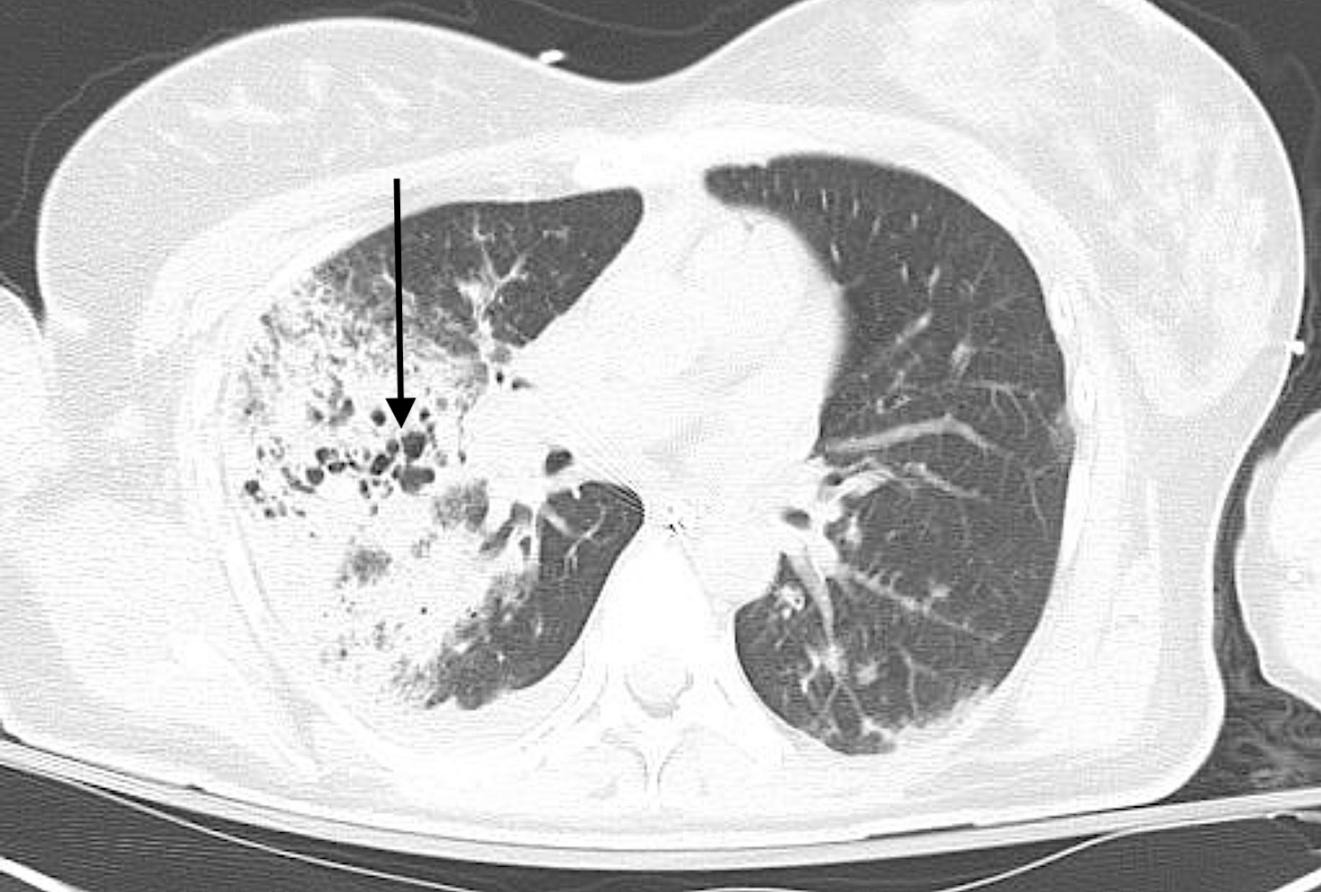
Computed tomography (CT) chest (axial view) showing cystic destruction of the lung parenchyma (arrow).

She was started on vancomycin, piperacillin-tazobactam and levofloxacin, and was transferred to the intensive care unit (ICU). Broad spectrum antibiotic therapy was chosen due to patient's critical condition and unclear etiology of the infecting pathogen. The patient had a white cell count of 11.8 x 10^3^/microliter (µL) that trended to 2.2 x 10^3^/µL, normal troponins, and a lactic acid of 1.8 mmol/Liter (L) initially that trended up to 2.5 mmol/L. Urine drug screen was positive for cocaine, benzodiazepine, and opiates. Urine Legionella antigen and *Streptococcus pneumoniae* antigen were negative. Influenza A was positive on rapid antigen testing and the patient was started on oseltamivir. This raised our concern for secondary bacterial pneumonia from MRSA as a complication of influenza virus infection. The patient had computed tomography (CT) of the chest that showed right upper, middle, and lower lobe consolidations with cystic destruction of the lung parenchyma (Figures 2, 3).

**Figure 4:**
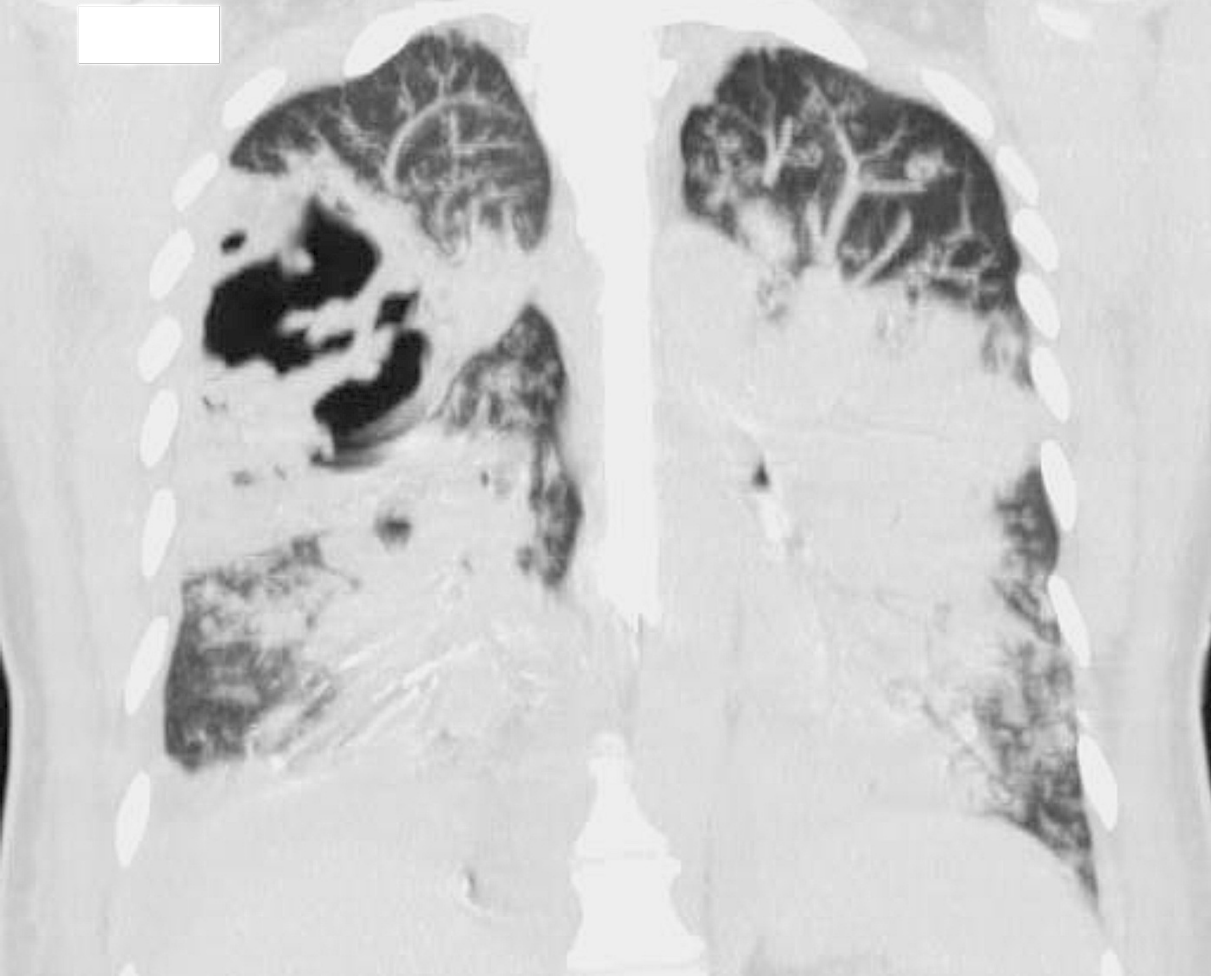
Repeat computed tomography (CT) chest (coronal view) showing worsening bilateral pneumonia.

**Figure 5:**
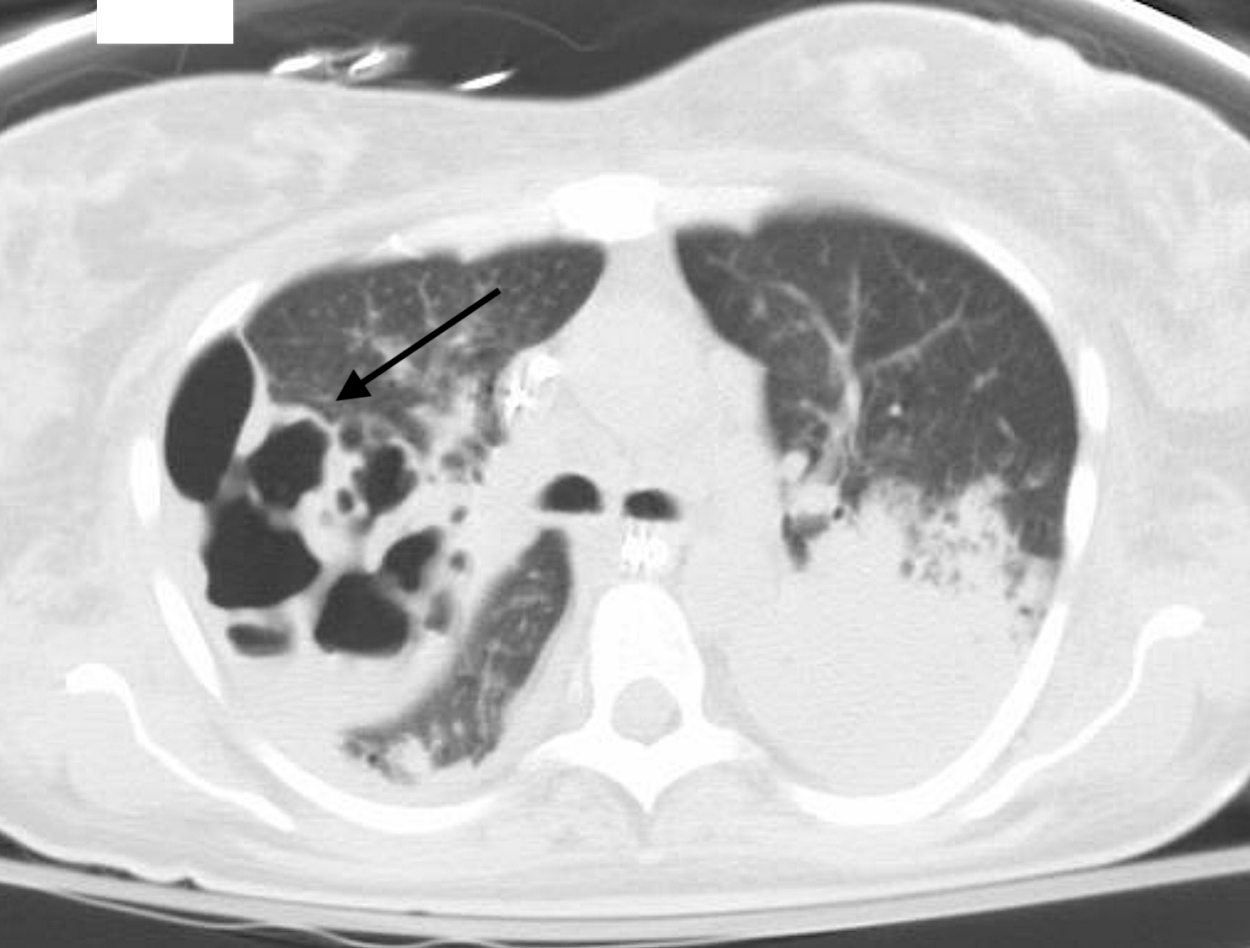
Computed tomography (CT) chest (axial view) showing worsening and multiple cavitary lesions in the right lung (arrow).

Sputum culture and blood cultures grew MRSA that was sensitive to both vancomycin and clindamycin, confirming our diagnosis. Vancomycin had already been started on admission and was continued. Fluid resuscitation was also done early in the course, but eventually progressed to septic shock. The patient was then started on norepinephrine infusion and vasopressin infusion for blood pressure support. She then developed acute respiratory distress syndrome (ARDS). Her hypoxia became refractory to conventional ventilation, and extracorporeal membrane oxygenation (ECMO) was considered. She had a transesophageal echocardiogram which showed severe biventricular systolic dysfunction, global hypokinesis with an ejection fraction (EF) of 5%. Her cardiomyopathy was felt to be acute and related to sepsis. She was managed for septic and cardiogenic shock, and venous-arterial ECMO was started. She was also started on continuous renal replacement therapy (CRRT) for hypervolemia refractory to diuresis, inotropic and vasopressor support. A follow-up transthoracic echocardiogram (TTE) five days later showed that the EF recovered to 45%. She continued to remain intubated for 16 days and a repeat CT chest showed worsening and multiple cavitary lesions in the right lung (Figures 4, 5).

A repeat TTE showed EF of 60% and normal left and right heart function. ECMO was stopped and she was decannulated on day 9, CRRT was stopped on day 9. Unfortunately, she did not tolerate withdrawal of ECMO and continued to deteriorate and became difficult to wean from the ventilator. Given her lack of response to vancomycin, she was switched to daptomycin and linezolid, and the vancomycin was discontinued. She did not improve with new antibiotic regimen, continued to accumulate volume, and could not tolerate being off CRRT that was resumed for volume removal. Her hypoxia continued to be refractory despite all efforts. The family eventually decided to transition to comfort care due to her poor prognosis.

## Discussion

MRSA can cause community-acquired pneumonia, and needs to be considered in the at-risk group of patients. This includes patients with known colonization with MRSA or those who have risk factors for colonization with MRSA (e.g., end-stage renal disease, contact sport participants, injection drug users, those living in crowded conditions, men who have sex with men, prisoners), recent influenza virus infection and antimicrobial therapy particularly with a fluoroquinolone in the prior three months. Also, it needs to be considered in community-acquired pneumonia presenting with necrotizing or cavitary lesions as in our patient, or with empyema. MRSA coverage should also be considered in community-acquired pneumonia severe enough to cause septic shock or respiratory failure requiring mechanical ventilation.

The most common CA-MRSA strain is USA300 [3]. Its pathogenicity appears to be greatly influenced by the Panton-Valentin-Leukocidin (PVL) toxin that causes leukocyte destruction, and also appears to play a major role in necrotizing pneumonia [3, 4]. The 2016 Infectious Disease Society of America recommendations regarding community-acquired pneumonia caused by MRSA or healthcare-associated pneumonia caused by MRSA is to treat with intravenous (IV) vancomycin or linezolid 600 milligrams (mg) twice daily either IV or oral [5]. While vancomycin has traditionally been used empirically for MRSA, it might have inadequate concentration in the lung tissue [6], tends to be under dosed in chronic kidney disease, might cause nephrotoxicity especially when combined with piperacillin-tazobactam, and it does not inhibit toxin production which is an important virulent factor in CA-MRSA pneumonia. On the other hand, linezolid appears to have greater lung penetration than vancomycin [3], does not need dose adjustment in patients with kidney disease, has great oral bioavailability and can be switched to oral regimen, and inhibits toxin production. Linezolid is the first of the oxazolidinone antibiotic that works by binding to the 50S ribosomal subunit, resulting in the inhibition of protein synthesis. Linezolid appears to have activity against not just the PVL toxin, but against several additional toxins including alpha-hemolysin and alpha-type phenol soluble modulins, which contribute to its virulence [3]. Caution should be exercised with using linezolid given its association with myelosuppression, neurotoxicity, and serotonin syndrome, especially with patients on selective serotonin reuptake inhibitors, as well as lactic acidosis with high dose or prolonged therapy [7]. Some prospective randomized trials showed that linezolid is superior to vancomycin in regards to clinical and microbiological response in both nosocomial and community MRSA pneumonia [1]. One of the concerns regarding the use of linezolid would be the selective pressure towards linezolid-resistant *Staphylococcus aureus*. Therefore, it is important for clinicians to identify risk factors for MRSA as well as the patient’s presentation. It is important to narrow the spectrum of antibiotic once susceptibility has been obtained, but it is just as important to monitor the patient’s disease progression. Additional considerations include the use of molecular biology testing to allow rapid identification of MRSA in the sputum. One study has shown that the test helped to reduce the use and cost of anti-MRSA antibiotics in patients suspected of ventilator-associated pneumonia [8].

## Conclusions

Accumulating body of evidence is suggesting that linezolid is superior to vancomycin in MRSA pneumonia in different clinical settings in terms of clinical response and microbiological clearance. However, there was no mortality benefit. Even though our patient was eventually switched to linezolid, unfortunately it was very late in the course. This case demonstrates the virulence of toxin-producing CA-MRSA, and consequent morbidity and mortality associated with CA-MRSA pneumonia. Given its high morbidity and mortality, clinicians might need to consider all measures that might help and as early as possible. We suggest using linezolid rather than vancomycin for CA-MRSA pneumonia, or at least adding a toxin inhibiting antibiotics in addition to vancomycin in the right clinical settings. We also suggest consideration of the diagnosis of CA-MRSA in patients with secondary bacterial pneumonia after influenza virus infection.
